# Assessing the feasibility of a web‐based outcome measurement system in child and adolescent mental health services – myHealthE a randomised controlled feasibility pilot study

**DOI:** 10.1111/camh.12571

**Published:** 2022-06-09

**Authors:** Anna C. Morris, Zina Ibrahim, Margaret Heslin, Omer S. Moghraby, Argyris Stringaris, Ian M. Grant, Lukasz Zalewski, Megan Pritchard, Robert Stewart, Matthew Hotopf, Andrew Pickles, Richard J. B. Dobson, Emily Simonoff, Johnny Downs

**Affiliations:** ^1^ South London and Maudsley NHS Foundation Trust London UK; ^2^ Department of Child and Adolescent Psychiatry, Institute of Psychiatry, Psychology and Neuroscience King's College London London UK; ^3^ Department of Biostatistics and Health Informatics, Institute of Psychiatry, Psychology and Neuroscience King's College London London UK; ^4^ NIHR South London and Maudsley Biomedical Research Centre London UK; ^5^ Health Service and Population Research Department, Institute of Psychiatry, Psychology and Neuroscience King's College London London UK; ^6^ Emotion & Development Branch, National Institute of Mental Health National Institutes of Health Bethesda MD USA

**Keywords:** Child and adolescent mental health, patient‐reported outcome measures, remote monitoring, acceptability

## Abstract

**Background:**

Interest in internet‐based patient reported outcome measure (PROM) collection is increasing. The NHS myHealthE (MHE) web‐based monitoring system was developed to address the limitations of paper‐based PROM completion. MHE provides a simple and secure way for families accessing Child and Adolescent Mental Health Services to report clinical information and track their child's progress. This study aimed to assess whether MHE improves the completion of the Strengths and Difficulties Questionnaire (SDQ) compared with paper collection. Secondary objectives were to explore caregiver satisfaction and application acceptability.

**Methods:**

A 12‐week single‐blinded randomised controlled feasibility pilot trial of MHE was conducted with 196 families accessing neurodevelopmental services in south London to examine whether electronic questionnaires are completed more readily than paper‐based questionnaires over a 3‐month period. Follow up process evaluation phone calls with a subset (*n* = 8) of caregivers explored system satisfaction and usability.

**Results:**

MHE group assignment was significantly associated with an increased probability of completing an SDQ‐P in the study period (adjusted hazard ratio (HR) 12.1, 95% CI 4.7–31.0; *p* = <.001). Of those caregivers' who received the MHE invitation (*n* = 68) 69.1% completed an SDQ using the platform compared to 8.8% in the control group (*n* = 68). The system was well received by caregivers, who cited numerous benefits of using MHE, for example, real‐time feedback and ease of completion.

**Conclusions:**

MHE holds promise for improving PROM completion rates. Research is needed to refine MHE, evaluate large‐scale MHE implementation, cost effectiveness and explore factors associated with differences in electronic questionnaire uptake.

**Key Practitioner Message**

• Patient‐reported outcome measures (PROMs) are considered an important tool for measuring treatment success and outcomes in healthcare systems.
• Adherence to routine PROM guidance in Child and Adolescent Mental Health Services (CAMHS) remains low, largely driven by limitations associated with paper‐based data collection.
• Paperless monitoring systems (i.e. digital) as an alternative to traditional outcome measure delivery and collection are growing in healthcare settings.
• Remote questionnaire completion using the myHealthE (MHE) system is feasible and acceptable to caregivers of children accessing CAMHS in South London. Results suggest a 12‐fold increase in Strengths and Difficulties questionnaire reporting compared to standard practice.
• More research is required to understand whether MHE implementation affords similar improvements in remote PROM completion at scale and whether electronic questionnaire uptake is equal for different socio‐demographic and clinical populations.

## Introduction

Patient‐reported outcome measures (PROMs) enable standardised and direct collection of a patient's perceived health status (Devlin & Appleby, 2010). Used routinely, PROMs are recognised as a clinically valuable method to measure patient‐ or caregiver‐rated symptoms, assess intervention success, and encourage shared patient and practitioner communication and decision making (Carlier, Meuldijk, Van Vliet et al., 2012; Lambert, Whipple, Hawkins et al., 2003; Soreide & Soreide, 2013). Child and Adolescent Mental Health Services (CAMHS) in England are encouraged to collect information about young people's presenting problems at entry to CAMHS and again within 6 months of receiving treatment (Department of Health (DoH), 2004, 2015; Morris et al., 2020) using PROMs. However, audit and survey studies demonstrate low guideline adherence, suggesting that CAMHS struggle to implement PROMs (Batty et al., 2013; Hall et al., 2013; Johnston & Gowers, 2005). Recent research investigating the electronic health records of 28,000 patients accessing CAMH services across South London identified paired use of the Strengths and Difficulties Questionnaire PROM (SDQ; Goodman, 1997), in only 8% of patients (Morris et al., 2020) and as few as 1% within specific clinical groups (Cruz et al., 2015).

Data collection using traditional paper questionnaires is associated with several time‐ and resource‐intensive steps, including printing, postage and processing returned outcome measures. Although paper questionnaires are practical and easy to complete, already‐burdened clinicians struggle with the administrative effort required to capture paper‐based questionnaires (Boswell, Kraus, Miller, & Lambert, 2015; Hall et al., 2014; Johnston & Gowers, 2005). Response data are also easily compromised, for example, users can omit questions, select multiple responses per item, and mark outside the questions tick box margins, leading to missing or unusable data (Ebert, Huibers, Christensen, & Christensen, 2018).

A rapid rise in internet use has paved the way for electronic questionnaires (Lyon, Lewis, Boyd, Hendrix, & Liu, 2016). Electronic PROMs (ePROMs) are reported to be less time consuming (Cella et al., 2015), require fewer administrative duties (Black, 2013; Coons et al., 2015; Eremenco, Coons, & Paty, 2014), cost less (Zuidgeest, Hendriks, Koopman, Spreeuwenberg, & Rademakers, 2011) and evoke more honest (Black & Ponirakis, 2000) and less erroneous responses; prompting patients to respond to all items within a questionnaire and only provide one response per question (Coons et al., 2015; Dillon et al., 2014; Eremenco et al., 2014; Jamison et al., 2001).

Feasibility trials of web‐based monitoring systems report positive outcomes relating to patient engagement, satisfaction and clinical value (Ashley et al., 2013; Barthel et al., 2016; Nordan et al., 2018; Schepers et al., 2017). However, less research is available on the development and application of ePROM systems in CAMHS. Interviews with mental health service users demonstrate positive attitudes toward the use of technology to assist traditional care (Borzekowski et al., 2009). However, patients have highlighted barriers to web‐based portal acceptability, including computer literacy, perceived usefulness, suitability, confidentiality, feedback and the effect application use has on their capacity to manage their condition and therapeutic relationships (Niazkhani, Toni, Cheshmekaboodi, Georgiou, & Pirnejad, 2020).

The myHealthE (MHE) system was built to enable remote PROM monitoring in CAMHS. This system aims to automate the communication, delivery and collection of ePROMs at predefined post‐treatment periods, providing caregivers with a safe and engaging way to share clinically relevant information about their child with their allocated care team with minimal human input. MHE architecture, development and implementation methodology, including key aspects of data safety and governance, have been described previously (Morris et al., 2021). MHE external web‐development was provided by Digital Marmalade (see Acknowledgements). Novel healthcare applications require feasibility and acceptability testing to ensure that the technology is understandable and can be used successfully by the target end‐user in real‐world clinical surroundings before conducting a large‐scale system evaluation (Steele Gray et al., 2016). As described in our protocol [(ISRCTN) 22581393], the primary purpose of this trial was to understand whether MHE use should be assessed in CAMHS on a wider scale. Therefore, we conducted a feasibility pilot study to evaluate whether introducing MHE increased completion of PROMS over the course of CAMHS treatment compared to standard data collection procedures, as measured by the proportion of ePROMS relative to paper questionnaires completed over a 3‐month period. Secondly, we aimed to assess caregiver satisfaction with the MHE system via individual caregiver phone consultations. Given resource constraints we were unable to assess the economic benefit of MHE compared to standard data acquisition as per our protocol. We hypothesised that MHE implementation would afford a substantial increase in completed standardised caregiver‐reported follow‐up data and caregiver satisfaction with CAMHS services compared to routine data collection.

## Methods

### Design

The current study comprised a single‐blindxed parallel group feasibility pilot randomised control trial (RCT) of MHE. Outcome, sociodemographic and service level data were obtained from the Clinical Record Interactive Search (CRIS) system. CRIS contains de‐identified medical record history from the South London and Maudsley (SLaM) National Health Service Foundation Trust, one of Europe's largest mental health care organisations providing services to over 34,400 children and adolescents between the 1 January 2008 and 1 December 2019 (Downs et al., 2019; Perera et al., 2016; Stewart et al., 2009). This research tool was established by SLaM's National Institute of Health Research Biomedical Research Centre (NIHR BRC) in 2008, to enable information retrieval for the purpose of approved research (Fernandes et al., 2013). Comprehensive electronic health record (EHR) information is available for SLaM services from 2006.

### Setting and participants

The trial was conducted at Kaleidoscope, a community paediatric mental health centre, based in Lewisham, South London, between the 11 February 2019 and the 14 May 2019. Eligible participants were caregivers of CAMHS patients aged between 4 and 18 years old with a diagnosis of autism spectrum disorder (ASD). Patients were under the care of Lewisham Neuro‐developmental Team and had at least one SDQ present in their EHR. Caregivers were recruited if they had contact details (mobile phone number and/or email address) recorded in their child's EHR. The MHE data collection process was directly comparable to current paper‐based practice, except for its electronic basis and only collected data which was ordinarily requested from families by their treating clinical team. Caregivers did not have to provide informed consent to participate in this trial, but could choose to opt‐out via email or phone call to the trial research assistant (ACM). Recruitment was achieved through SLaM EHR screening. A Microsoft SQL script was developed and implemented by a senior member of the SLaM Clinical Systems Team and automatically provided an extract of eligible patients to the research team. Subsequently, computerised condition allocation and simple randomisation assigned eligible caregivers to either receive PROM outcome monitoring as usual (MAU; control group) or enrolment to the MHE platform (intervention group) on a 1:1 basis. Clinicians were blinded to condition allocation, and not informed which patients on their case load had been allocated to receive MHE or MAU.

### Measures, sociodemographic and clinical characteristics

The primary outcome variable was time to completed follow‐up caregiver SDQ (SDQ‐P; electronic vs. paper SDQ‐P) within the 3‐month observation period. The SDQ‐P (Appendix 1) is a structured 25‐item questionnaire screening for symptoms of childhood emotional and behavioural psychopathology (Goodman, 1997). SLaM holds a sub‐licence to use the SDQ to support clinical service via NHS Digital Copyright Licensing Service. It is current clinical practice to collect SDQ‐P for young people, either by post before their first face‐to‐face meeting, or on site during a clinical appointment to inform their baseline assessment and again 6 months after starting treatment or upon discharge from CAMHS. Other variables extracted from CRIS are presented in Table S1.

### Process evaluation: usability testing

To evaluate MHE usability, we contacted by telephone a subset of caregivers randomly assigned to MHE. This subset included a convenience sample of six caregivers who had engaged with MHE and two caregivers who had not. Caregivers were asked to access the MHE portal and complete the System Usability Scale (SUS; Brooke, 1996) to examine subjective usability. SUS comprises 10 statements reported on a 5‐point Likert scale ranging from strongly disagree to strongly agree. The total score is presented as a figure from 0 to 100, with a greater score reflecting higher usability. Mean SUS score was computed and ranked using Bangor, Kortum, and Miller's (2008) acceptability scale defined as ‘not acceptable’, ‘marginal’ and ‘acceptable’. Following administration of the SUS, caregivers were invited to ask questions about the platform or provide any further comments about their experience of using MHE.

### Sample size

The current trial aimed to inform the development of a larger, adequately powered RCT by providing precise estimates of acceptability and feasibility, in addition to outcome variability. A threshold of clinical significance was decided a priori to be 15% between MAU and MHE groups for SDQ‐P completion within 3‐months, based on consensus from Kaleidoscope staff and previous research indicating an expected baseline completion rate of 8% SDQ‐P in the control group (Morris et al., 2020). For a fixed sample size design, the sample size required to achieve a power of 1 − β = .80 for the two‐tailed chi‐square test at level α = .05, under the prior assumptions, was 2 × 91 = 182 on a 1:1 allocation ratio. The power calculation was carried out using Gpower 3.1.7. To increase power and reduce the risk of chance imbalance between MHE and non‐MHE groups, we followed recent guidance on covariate adjustment within RCTs of moderate sample size (Kahan, Jairath, Doré, & Morris, 2014, and included in our analyses, several factors which could have potential influence on PROM completion (Morris et al., 2020).

### Intervention and procedure

**Figure 1:**
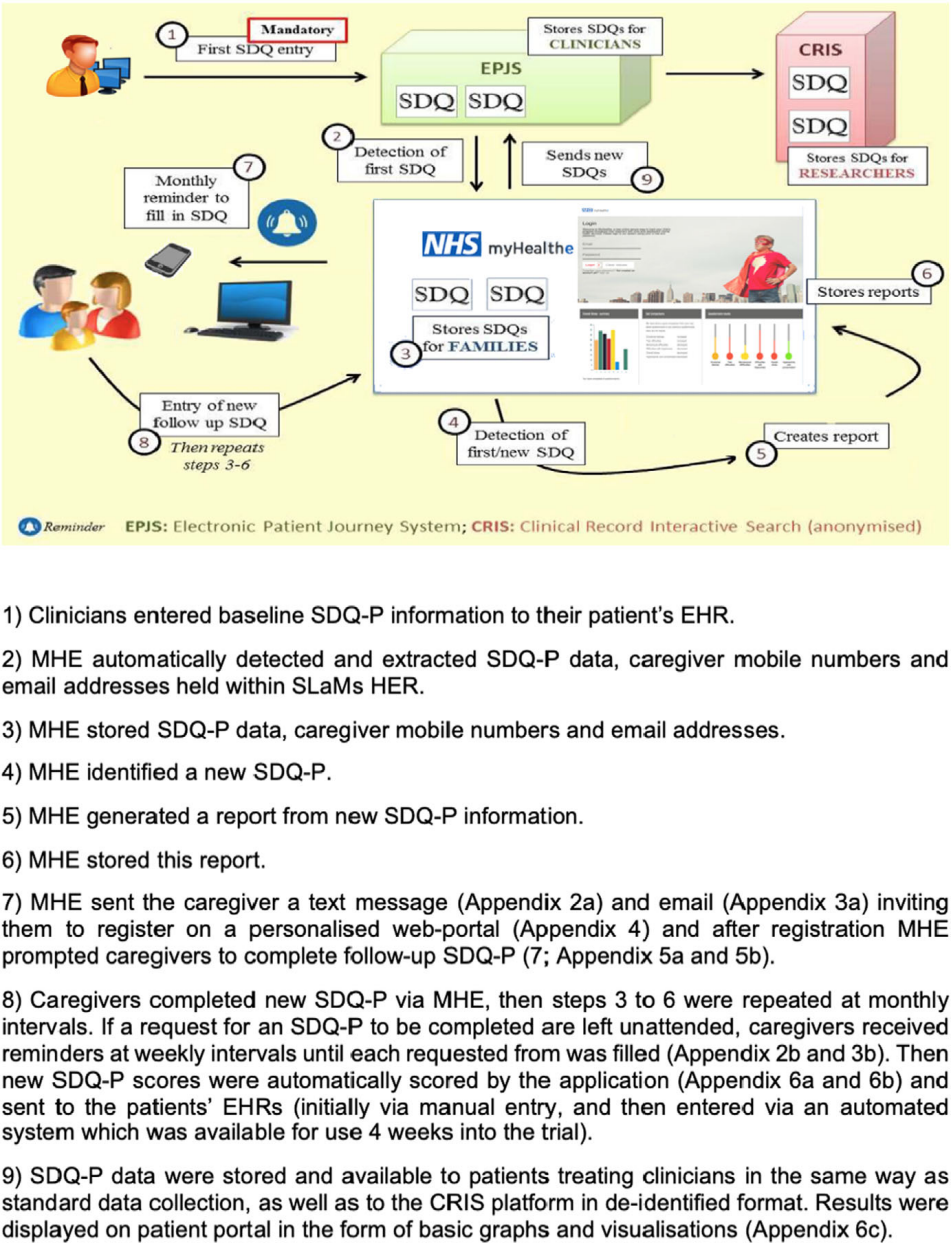
myHealthE data flow‐diagram

Figure 1 provides an overview and description of the MHE data flow. All caregivers of patients receiving care from Lewisham Neurodevelopmental Team were contacted by letter. This letter informed them of potential changes to clinical information collection (i.e. electronic rather than paper questionnaires) and provided with an information sheet and MHE information leaflet (Appendix 7a,b). After group assignment, caregivers allocated to receive MHE were contacted with a text (Appendix 2a) or email message (Appendix 3a) inviting them set up a personalised web‐portal (Appendix 4) and complete an SDQ‐P (Appendix 5a,b, caregivers were enrolled in the trial irrespective of whether they registered their MHE account). Caregivers who did not register were sent an automated weekly prompt to enrol and complete an SDQ‐P (see Appendix 2b and 3b). Once an online questionnaire was completed, caregivers were presented with infographics based on their responses (Appendix 6a–c), and they were then contacted monthly to provide follow‐up SDQ data. In the control group caregivers were requested to complete paper SDQ‐P face‐to‐face or by post according to clinician discretion. Apart from electronic SDQ‐P completion for the intervention group, treatment remained the same for all participants. Information collected through MHE was stored in the child's EHR and managed in the same way as all other confidential information. SDQ‐P data were checked daily by ACM and promptly entered to the patient's EHR. Post intervention, all participants received a letter thanking them for their participation.

### Strategy for analysis

All analyses were conducted using STATA version 14 (StataCorp., 2015). Analyses were conducted to determine differences in SDQ‐P completion between paper based (MAU, monitoring as usual) approaches and MHE. Analysis was performed subject to intention‐to‐treat like principles (intention‐to‐contact), whereby all participants were analysed according to their initially assigned intervention arm, irrespective of protocol adherence or deviations. Cox regression was used to examine the relationship between MAU versus MHE group assignment and SDQ‐P completion rates. Using a Kaplan–Meier curve, we checked whether group assignment (as predictor) satisfied the proportional hazards assumption. Our first analysis examined the association between treatment group only and SDQ‐P completion. The second model adjusted for demographic and clinical covariates captured in this trial. An inverse Kaplan–Meier curve was plotted to visualise the probability of SDQ‐P completion, comparing caregivers who completed electronic and paper SDQ‐P. For the intervention group the MHE website–SDQ‐P completion conversion rate was reported as a percentage by measuring the number of caregivers that register on MHE and subsequently completed a follow‐up SDQ‐P.

## Results

### Enrolment and baseline characteristics

**Figure 2:**
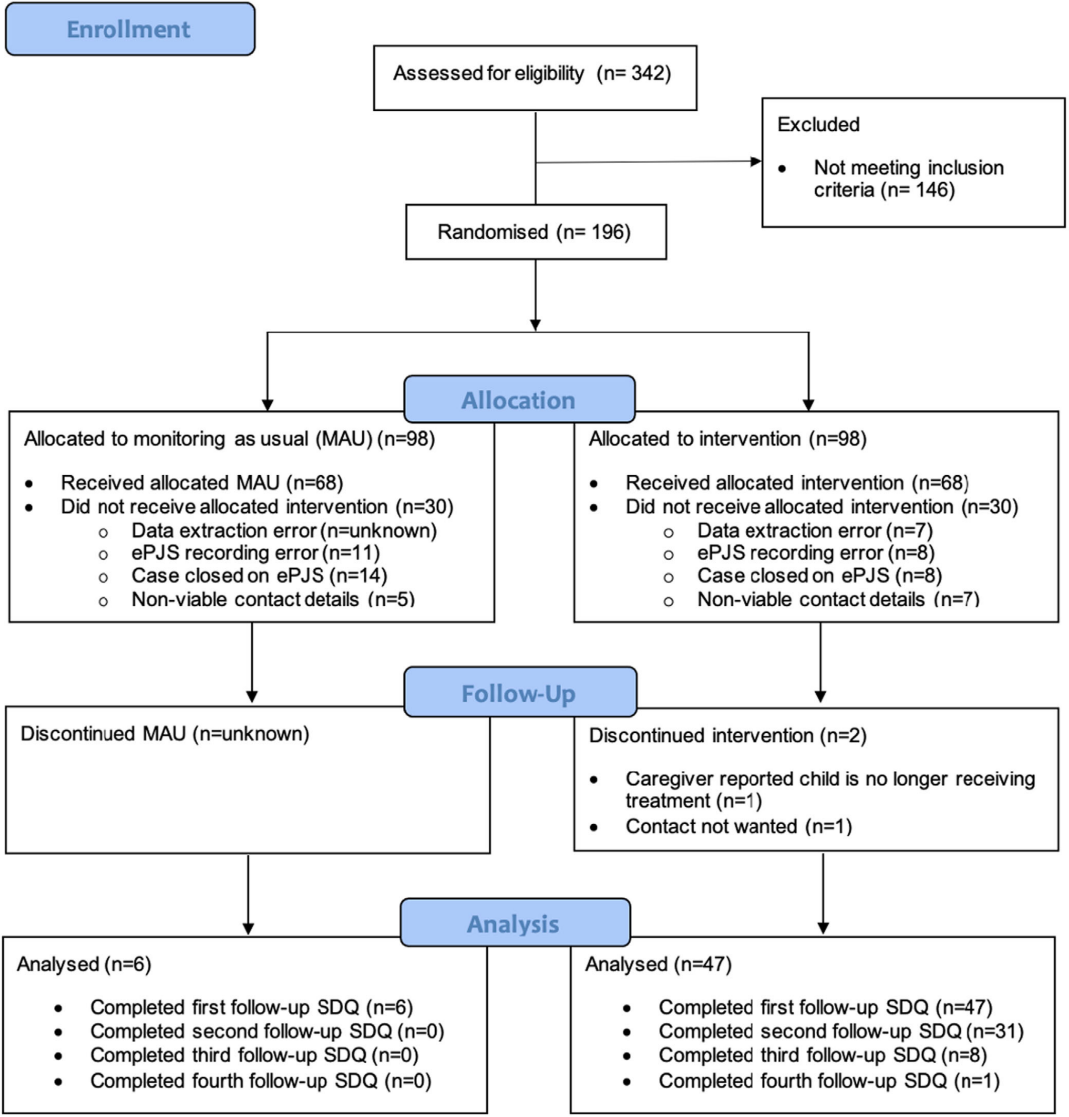
Consort diagram presenting recruitment and rate of data collection for MHE and MAU

Within study, participant flow and data collection rates are provided in Figure 2. A total of 342 caregivers were screened for eligibility of which (*n* = 196) met the inclusion criteria. Of the 146 excluded, the majority were due to lack of baseline SDQ (*n* = 132) During eligibility screening caregiver contact information was often missing or located in an area of the patients' EHRs different from expected, therefore manual contact detail collection was carried out to enable digital communication via MHE. In some cases, no current parental mobile phone number nor email address was found within the EHR (*n* = 14). Caregivers were enrolled and randomly assigned to the intervention group (MHE *n* = 98) and the control group (MAU *n* = 98). Of caregivers assigned to MHE and MAU, 30 (36.3%) did not receive notifications from MHE, with the text monitoring system logging these mobile numbers were incorrect or not in use The conversion rate from account registration to SDQ completion was 98% (47/48). Table S2 outlines account registration issues and opt‐out preferences reported by caregivers.

Table 1 presents sociodemographic and service characteristics for the whole sample. Participants were ethnically diverse, predominantly male and at the older end of the age range accepted by CAMHS.

**Table 1:** Baseline patient and clinical characteristics of MHE versus MAU following randomisation (*n* = 196)

	Total sample *n* = 196	
	MAU = 98	MHE *n* = 98
Gender, *n* (%)		
Male	74 (75.5)	74 (75.5)
Mean age at trial start (SD)	14.3 (2.7)	14.3 (2.8)
Ethnicity, *n* (%)		
White	39 (39.8)	46 (46.9)
Black	35 (35.7)	23 (23.5)
Asian	2 (2.0)	1 (1.0)
Mixed	13 (13.3)	15 (15.3)
Other or not stated	9 (9.2)	13 (13.3)
Level of deprivation, *n* (%)		
1st (least deprived)	22 (23.2)	25 (25.8)
2nd	21 (22.1)	27 (27.8)
3rd	24 (25.2)	24 (24.7)
4th (most deprived)	28 (29.5)	21 (21.7)
Co‐morbid diagnosis, *n* (%)		
ADHD	48 (49.0)	39 (39.8)
LD	14 (14.3)	11 (11.2)
Emotional disorder	14 (14.3)	17 (17.4)
Mean CGAS score (*SD*)	53.1 (10.7)	54.6 (8.8)
Mean days of active care (*SD*)	592.1 (196.4)	563.0 (210.4)
Mean attended F2F events (*SD*)	6.0 (10.2)	9.1 (21.0)
Mean baseline SDQ Scores (*SD*)		
Emotional	5.3 (2.5)	5.5 (2.8)
Conduct	4.3 (2.3)	4.5 (2.4)
Hyperactivity	7.5 (2.3)	7.7 (2.1)
Peer difficulties	4.8 (2.3)	5.2 (2.3)
Prosocial	5.2 (2.6)	5.4 (2.3)
Impact score	5.9 (3.4)	5.6 (2.9)
Total difficulties	22.1 (5.5)	23.0 (5.3)

*SD*, standard deviation.

### Electronic versus paper SDQ‐P collection

**Figure 3:**
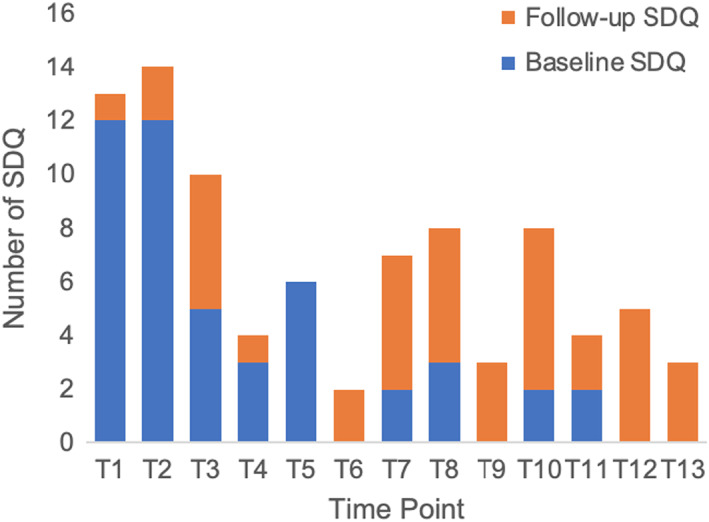
Baseline and follow‐up SDQ completion within each 7‐day notification period

During the trial 47 caregivers [47.9% of intention‐to‐contact (total *n* = 98), 69.1% of actually contacted (total *n* = 68)] registered an account on the MHE platform and completed at least one follow‐up SDQ‐P. In the corresponding timeframe 6 (intention to contact = 6% (*n* = 98) and actually contacted = 8.8% (*n* = 68) caregivers assigned to receive MAU completed at least one follow‐up SDQ‐P. Second follow‐up was due for 43 of the MHE cohort by the end of the study period (at least 1 month had elapsed since completing their first online SDQ‐P) and of these 31 caregivers completed this (72%). Overall, 87 follow‐up SDQ‐Ps were completed via the MHE platform: Figure 3 provides a breakdown of SDQ‐P completion within each 7‐day notification reminder period.

**Figure 4:**
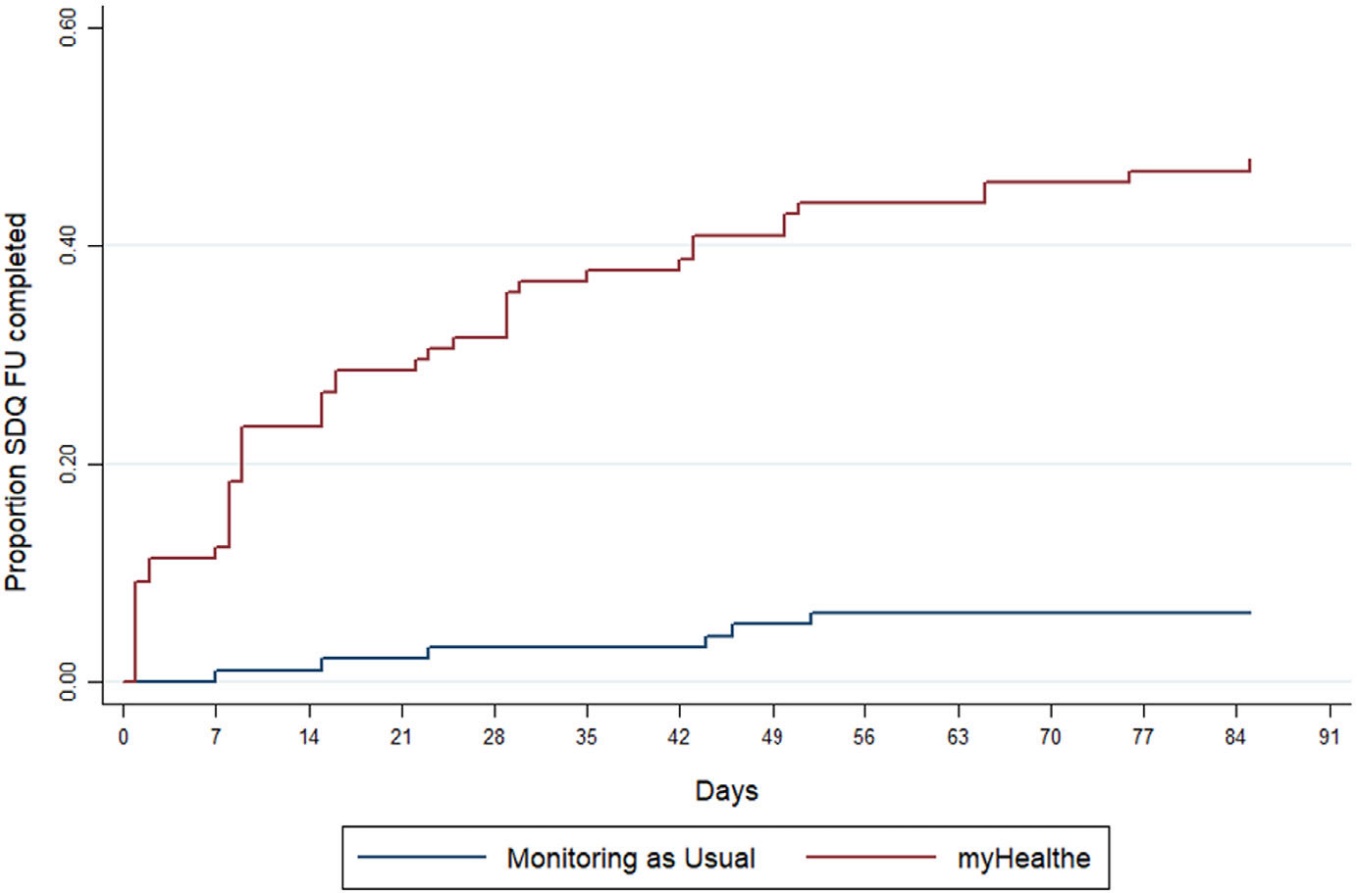
Kaplan‐Meier curve illustrating the probability of SDQ‐P within study period between caregivers assigned to complete electronic compared to paper SDQ‐P

The ITC Cox regression models are presented in Table 2, and graphically depicted in Figure 4. MHE group assignment was significantly associated with an increased probability of completing an SDQ‐P in the study period (adjusted hazard ratio (HR) 12.1, 95% CI 4.7–31.0; *p* = <.001). This was observed after controlling for potentially confounding socio‐demographic characteristics and clinical factors including, gender, age at the start if the trial, baseline CGAS (Schaffer et al., 1983) and SDQ profiles, co‐morbid ADHD, learning disability, and emotional disorders as well as number of days of active care and attended face‐to‐face events. No significant interaction was found between ethnic status (white and non‐white ethnic groups) and SDQ‐P completion by group.

**Table 2:** An Intention to contact Cox‐regression analysis of the relationship between electronic compared to paper‐based SDQ‐P assignment and SDQ‐P completion rates (*n* = 195), adjusted model taking into account participant characteristics

	Crude H.R (95% CI)	*p*‐Value	Adjusted model H.R (95% CI)	*p*‐Value
Group (MHE vs. MAU)	10.1 (4.3–23.6)	<.01	12.1 (4.7–30.9)	<.01
Gender				
Male			0.4 (0.2–0.8)	.02
Ethnicity				
White			Reference	–
Black			0.5 (0.2–1.2)	.13
Asian^a^			na	na
Mixed			1.1 (0.4–2.5)	.88
Other or not stated			0.5 (0.2–1.4)	.16
Age at trial start			1.0 (0.9–1.1)	.90
Co‐morbid diagnosis				
ADHD			0.8 (0.4–1.6)	.47
LD			1.5 (0.6–4.0)	.44
Emotional disorder			2.5 (1.0–5.8)	.04
Days of active care			1.0 (1.0–1.0)	.77
Attended F2F events			1.0 (1.0–1.0)	.61
Baseline SDQ scores				
Emotional			1.0 (0.9–1.2)	.61
Conduct			1.0 (0.9–1.1)	.85
Hyperactivity			1.1 (1.0–1.3)	.17
Peer difficulties			1.0 (0.9–1.2)	.94
Prosocial			1.1 (0.9–1.2)	.94

^a^ Covariate dropped due to <5 cell size value.

### Caregiver perspective of MHE implementation

**Figure 5:**
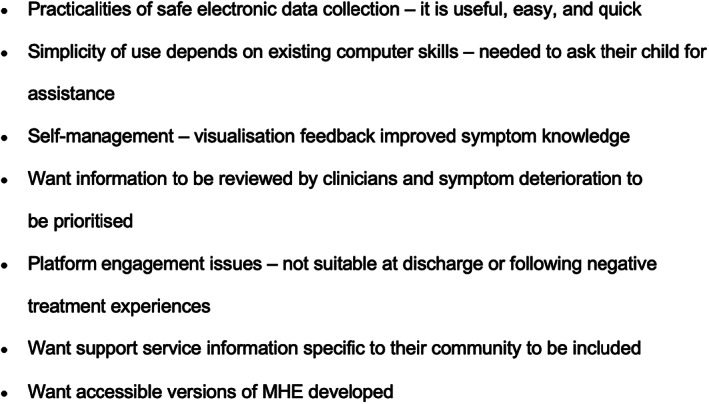
Summary of patient feedback following MHE use

A total of eight SUS questionnaires and usability interviews were completed. The mean SUS score for users of the website was 78/100 indicating that the application was ‘acceptable’ to users. Figure 5 provides a summary of caregiver's comments regarding MHE.

## Discussion

This feasibility pilot showed that the collection of electronic PROMs using web‐based technology is feasible in CAMHS practice. Implementation of MHE, a novel remote monitoring platform afforded considerable rates of SDQ‐P completion (69%) for caregiver's who received an invitation to register for MHE compared to 12% paper‐based SDQ‐P completion. By way of contrast, a comprehensive audit of over 28,000 young people services accessing CAMHS found paired SDQ‐P completion rates of 8%. By automating unassisted delivery of PROMs at specified time points, MHE may address several fundamental challenges inherent to paper‐based information gathering in busy clinical settings, such as processing burden, lack of supportive infrastructure and poor administration guideline knowledge (Boswell et al., 2015; Duncan & Murray, 2012; Waldron, Loades, & Rogers, 2018; Wolpert, 2014).

In post‐trial interviews caregivers rated MHE as ‘acceptable’, suggesting good levels of usability. Many caregivers favoured the ease and speed of using MHE to complete outcome measures compared to paper‐based methods, while barriers included how readily information provided through the platform was used by clinicians to identify children with worsening symptoms and data privacy concerns. However, only a small number of caregivers were contacted to provide their views on the system; therefore, it is possible that other undetected usability issues influenced the results of this trial, for example: language, literacy level, disability, and cultural sensitivity difficulties (Bodie & Dutta, 2008; Kontos, Bennett, & Viswanath, 2007; Lindsay, Bellaby, Smith, & Baker, 2008; Morey, 2007).

Historically, low engagement with eHealth has been attributed to unequal internet access (Latulippe, Hamel, & Giroux, 2017) but did not appear to account for non‐engagement in the current trial. This finding is likely to reflect the substantial increase in mobile phones and other internet‐enabled mobile technology availability (Pew Research Center, 2019), reduced cost of internet subscriptions and widening availability of free public Wi‐Fi (Kontos et al., 2007; McAuley, 2014). However, despite physical internet access, end‐users may not have the skills necessary to fully engage with digital technologies (Hargittai, 2002). This was the case for several caregivers who reported that their limited information technology capabilities and knowledge, making it hard to navigate MHE without assistance from family members. This disparity may deepen as digital platforms are increasingly integrated into routine clinical practice (Van Dijk, 2005) and should be iteratively considered during the design and implementation of emerging digital health platforms, paying particular attention to the role of co‐design (Andersen, 2019).

### Strengths and limitations

This trial was conducted in a naturalistic manner independent of clinical practice to ensure that clinician's behaviour, for example, promoting MHE use did not inflate observed rates of engagement. Moreover, the research was conducted in a socio‐demographically diverse geographical area, resulting in a broad range of caregivers testing the system. Finally, condition allocation was computerised meaning that all participants were instantly allocate to either receive MAU or MHE. Therefore, it was unlikely that allocation bias would have influenced the trial findings.

Limitations include the fact that families only had the opportunity to enrol to the trial if they had a baseline SDQ present in their child's EHR, which relies on this being initiated by a clinician in the first instance. In the future, using MHE to capture baseline and follow up SDQ‐P data may afford a more realistic assessment of ePROM feasibility. It is also possible that neurodevelopmental team service users perceived the SDQ‐P as less useful than a disorder specific questionnaire, which may have resulted in lower rates of completion level. As we were primary focused on developing an interface for parents, co‐design sessions with clinicians were limited. Further work is needed to examine what is potentially lost using ePROMS compared pencil and paper approaches, and how this could be mitigated by improved design within later versions of myHealthE. Lastly, owing to resource constraints phone interviews were conducted after the trial ended meaning that responses could be influenced by recall bias.

### Future research and MHE refinement

The next phase of this research is to extend this feasibility study across multiple‐healthcare sites and other child mental health specialties and additional pertinent PROMs. Plans are already in place to extend MHE introduction to national and specialist teams and further SLaM CAMHS teams across Southwark, Lambeth and Croydon. Recent funding secured from the National Institute for Health Research (NHIR; https://fundingawards.nihr.ac.uk/award/RP‐PG‐0618‐20003) and the Medical Research Council (MRC) Mental Health Pathfinder award to King's College London has enabled MHE to be converted into a scalable NHS software as a service (SaaS) product, with a roadmap to implement MHE across four other Trusts in England. Collecting data from a larger number of caregivers will enable us to explore the effects of various patient factors on ePROM engagement. Research investigating differential uptake in PROM collection suggests that several patient characteristics including ethnicity and social deprivation are associated with inequitable PROM use (Latulippe et al., 2017; Morris et al., 2020). While this was not the case in the current small‐scale trial, it is essential that further research is conducted to determine whether these systems sustain possible health inequalities with larger sample sizes. System refinements are also required to enable alternative methods for acquiring and inputting caregiver contact information to circumvent the difficulties encountered with automatic data extraction in this study.

In‐depth interviews are needed to explore how ePROM platforms can be adapted to meet different service user and clinician needs. Qualitative work is needed to provide more general insights into: (a) caregivers' reasons for deciding to complete or not complete electronic questionnaires; (b) clinicians' perspectives on how digital collection systems and analysis of outcomes could enhance decision making at individual level; (c) clinician and caregivers' views on the concept, design and delivery of MHE, the barriers and facilitators for MHE implementation and identify potential harms and study protocol refinement (e.g., platform design and frequency of questionnaire completion); and (d) young people's perspective on whether the MHE could be adapted as self‐reported outcome collection system, and if trialled, how it should be evaluated.

## Conclusion

Routine PROM collection is essential for delivering personalised health services that reflect clinical need from the perspective of young people and their families. This study supports the feasibility of a remote PROM monitoring platform within a real‐world outpatient setting providing treatment to a demographically diverse population. Intimating that web‐platforms may provide an acceptable and convenient method to maintain and scale up improved patient monitoring, service‐user communication, and service evaluation. A future multisite trial of MHE is required to evaluate this e‐system at scale.

## Ethical information

Approval for the study was given by the South London and Maudsley NHS Foundation Trust CAMHS Clinical Audit, Service Evaluation and Quality Improvement Committee (approval date: 07/04/2017). Extraction and analysis of deidentified outcome data were carried out using the CRIS platform and security model approved by Oxford Research Ethics Committee C (reference 18/SC/0372).

**Trial Registration:** International Standard Randomised Controlled Trial Number (ISRCTN) 22581393; https://doi.org/10.1186/ISRCTN22581393.

## (b) Electronic Strengths and Difficulties Questionnaire

## (b) Strengths and Difficulties Questionnaire previous results summary

## (c) Strengths and Difficulties Questionnaire results visualisation

## Supporting information


**Table S1.** List of socio‐demographic and clinical variables extracted from CRIS.
**Table S2.** Description of caregiver opt‐out preferences and technical difficulties encountered at MHE registration.
