# Supplementary material for: Assessing the feasibility of a web‐based outcome measurement system in child and adolescent mental health services – myHealthE a randomised controlled feasibility pilot study
Source: Child Adolesc Ment Health. 2022 Jun 9;28(1):128–47. doi: 10.1111/camh.12571 (PMC10083915; doi:10.1111/camh.12571)
Supplement: Supplementary file 1 — Table S1. List of socio‐demographic and clinical variables extracted from CRIS. Table S2. Description of caregiver opt‐out preferences and technical difficulties encountered at MHE registration. [file CAMH-28-128-s001.docx]

**Supporting Information**

**Table S1.** List of socio-demographic and clinical variables extracted from CRIS.

| **CRIS variable** | **Description** |
| --- | --- |
| Gender | Gender of the child recorded in CRIS at the trial start date. |
| Age | Age of the child recorded in CRIS at the trial start date. |
| Ethnicity | Child’s ethnicity was collapsed from source EHR codes into five broad categories (as defined by the UK Office for National Statistics): 1) White (White British, Irish and Other White Background), 2) Black (African, Caribbean and Other Black), 3) Asian (Indian, Pakistani, Bangladeshi, Chinese and Other Asian), 4) Mixed and Other (White and Black Caribbean, White and Black African, White and Asian and Other Mixed and any other ethnic group), and 5) Not stated (ethnicity not provided). |
| Neighbourhood deprivation | An index of neighbourhood deprivation for the main caregiver residence comprised in the current sample, categorised into quartiles of multiple deprivation (McLennan, Barnes, Noble et al., 2011). |
| ICD-10 primary or secondary diagnosis | Child’s most recent ICD-10 primary or secondary diagnosis at the trial start date. |
| Children’s Global Assessment Scale (CGAS) | Most recent CGAS (Shaffer, Gould, Brasic et al., 1983) outcome, a clinician reported assessment of patient functioning reported as a single score from 0 (extremely impaired) to 100 (doing very well). |
| Active care days | Number of active care days (inpatient and outpatient) recorded in the two years before the trial start date. |
| Face-to-face events | Number of attended face-to-face CAMHS events recorded in the two years preceding the trial start date. |
| SDQ sub-scale scores | SDQ sub-scale scores for the most recently recorded SDQ before the start of the trial. |

**Table S2.** Description of caregiver opt-out preferences and technical difficulties encountered at MHE registration.

| **Platform engagement Issue** | **Reason** | **Outcome** |
| --- | --- | --- |
| Opt-out (n=2) | Patient 1: Felt it was unnecessarily complicated to set up an account on MHE and did not believe that using MHE would help their child receive quicker treatment.  Patient 2: Their child was no longer accessing CAMHS. | Patient 1: Successfully opted-out and received no further communication from MHE.  Patient 2: Successfully opted-out and received no further communication from MHE. |
| Technical difficulties (n=2) | Patient 1 & 2: MHE login page was not auto populated with the caregiver’s unique user ID, rendering users unable to set up their account. | Patient 1 & 2: Registration link was resent, and the issue desisted. |
